# A New Computational Model of High-Order Stochastic Simulation Based on Spatial Legendre Moments

**DOI:** 10.1007/s11004-018-9744-z

**Published:** 2018-06-04

**Authors:** Lingqing Yao, Roussos Dimitrakopoulos, Michel Gamache

**Affiliations:** 1 Department of Mathematics and Industrial Engineering, École Polytechnique, Montreal, QC H3T 1J4 Canada; 2 COSMO – Stochastic Mine Planning Laboratory, Department of Mining and Materials Engineering, McGill University, 3450 University Street, Montreal, QC H3A 2A7 Canada

**Keywords:** High-order stochastic simulation, Multi-point statistics, Spatial moments, Legendre polynomials

## Abstract

Multiple-point simulations have been introduced over the past decade to overcome the limitations of second-order stochastic simulations in dealing with geologic complexity, curvilinear patterns, and non-Gaussianity. However, a limitation is that they sometimes fail to generate results that comply with the statistics of the available data while maintaining the consistency of high-order spatial statistics. As an alternative, high-order stochastic simulations based on spatial cumulants or spatial moments have been proposed; however, they are also computationally demanding, which limits their applicability. The present work derives a new computational model to numerically approximate the conditional probability density function (cpdf) as a multivariate Legendre polynomial series based on the concept of spatial Legendre moments. The advantage of this method is that no explicit computations of moments (or cumulants) are needed in the model. The approximation of the cpdf is simplified to the computation of a unified empirical function. Moreover, the new computational model computes the cpdfs within a local neighborhood without storing the high-order spatial statistics through a predefined template. With this computational model, the algorithm for the estimation of the cpdf is developed in such a way that the conditional cumulative distribution function (ccdf) can be computed conveniently through another recursive algorithm. In addition to the significant reduction of computational cost, the new algorithm maintains higher numerical precision compared to the original version of the high-order simulation. A new method is also proposed to deal with the replicates in the simulation algorithm, reducing the impacts of conflicting statistics between the sample data and the training image (TI). A brief description of implementation is provided and, for comparison and verification, a set of case studies is conducted and compared with the results of the well-established multi-point simulation algorithm, filtersim. This comparison demonstrates that the proposed high-order simulation algorithm can generate spatially complex geological patterns while also reproducing the high-order spatial statistics from the sample data.

## Introduction

For the past several decades, stochastic simulations have been used to quantify spatial uncertainty in earth science applications. Traditionally, stochastic models are built on the basis of the Gaussian distribution and two-point statistics, where covariance or variograms are used to capture the spatial correlations (David 1988; Deutsch and Journel 1992; Journel 1994; Goovaerts 1997). The limitations of the existing two-point simulation methods have been reported in various publications (Guardiano and Srivastava 1993; Xu 1996; Journel 1997, 2003; De Iaco and Maggio 2011), which are mostly related to the poor reproduction of spatial distributions while dealing with the complex spatial patterns, spatial connectivity of extreme values, and non-Gaussianity. To reflect the complex geological patterns, multi-point statistics have to be introduced instead of conventional two-point statistics. Guardiano and Srivastava (1993) propose a multiple-point simulation (mps) framework and the concept of the training image (TI). The primary difference between mps and two-point simulations is that the conditional cumulative distribution functions (ccdfs) are built on empirical estimations of conditional probabilities with multiple-point configurations, which is equivalent to solving a normal equation according to the Bayes’ rule. Strebelle (2002) formalizes the method and developed the first computationally efficient implementation. For over a decade, research has been focused on various issues around mps algorithms, such as computational efficiency and various patch-based extensions (Zhang et al. 2006; Arpat and Caers 2007; Wu et al. 2008; Boucher 2009; Remy et al. 2009; Honarkhah and Caers 2010; Mariethoz et al. 2010; Parra and Ortiz 2011; Huang et al. 2013; Boucher et al. 2014; Strebelle and Cavelius 2014; Chatterjee et al. 2016; Li et al. 2016). In general, these mps methods are TI-based, and their statistics are estimated from distributions of replicates of data events in the TI. Their main drawbacks are: (1) the high-order statistics are partially and indirectly considered; (2) the methods are not driven by a consistent mathematical framework; and (3) since they are TI-driven, they may not generate results that comply with the statistics of actual available data. The latter shortcoming becomes distinctly clear in mining applications, where dense data sets are used (Osterholt and Dimitrakopoulos 2007; Goodfellow et al. 2012).

As an alternative, a high-order simulation framework with mathematical consistency is proposed with the introduction of a new concept of spatial cumulants (Dimitrakopoulos et al. 2010). The so-called high-order simulation algorithm (hosim) and its implementation are developed by Mustapha and Dimitrakopoulos (2010b, 2011). In this algorithm, the conditional probability density function (cpdf) is approximated by a multivariate expansion with coefficients expressed in terms of spatial cumulants. The hosim algorithm has been extended mostly recently to deal with the joint simulation of multiple variables, as well as the simulation of categorical data (Minniakhmetov and Dimitrakopoulos 2017a, b); other extensions are approximating the cpdf with different types of orthogonal polynomial bases, such as expansion series with Laguerre polynomials and Legendre-like spline polynomials (Mustapha and Dimitrakopoulos 2010a; Minniakhmetov and Dimitrakopoulos 2018). However, the related calculations are computationally demanding, since the number of spatial cumulants involved in the series increases exponentially either as the order of cumulants or the quantity of conditioning data increases. In Mustapha and Dimitrakopoulos (2011), some terms of the expansion series have to be discarded to obtain computational feasibility, which compromises the accuracy of the approximated cpdf. In addition, the computational cost limits the approach for larger-scale applications.

To take full advantage of the high-order simulation, that is, its data-driven aspect and no presumption of data distribution, and address the computational difficulties, a new stochastic simulation algorithm based on high-order spatial Legendre moments is presented herein. Rather than just a mathematical equivalency of the previous model of the high-order simulation, the approximation of the cpdf by Legendre polynomial series is reformulated under the framework of the sequential simulation, leading to a much more concise form of the computational model. In this new method, all explicit calculations of moments are encapsulated in a unified function to derive the cpdf, cutting down the previous complex computations into a few iterations of simple operations with polynomial time. Moreover, there is no predefined template configuration in the new algorithm, as required for the normal mps methods and the previous hosim model. The spatial configuration of the template will, instead, depend on the local neighborhood of the node to be simulated; note that there is no need to store the intermediate results in a tree as in most of the mps methods, including the previous hosim model. The variable template also has the advantage of simultaneously capturing the spatial patterns either on a local scale or a global scale.

The remainder of the paper continues with Sect. 2, which describes the stochastic model based on the concepts of high-order spatial Legendre moments. Section 3 develops the computational model as a statistical function. Section 4 describes the new proposed high-order simulation algorithm and analyzes the computational complexity. Section 5 explores the implementation of the new high-order simulation algorithm. Section 6 shows examples to assess the new method and compare it with filtersim. Finally, conclusions and future research are presented in Sect. 7.

## Stochastic Model of High-Order Simulation with Spatial Legendre Moments

### Sequential Simulation

In this paper, the stochastic model is discussed specifically under the sequential simulation framework (Rosenblatt 1952; Johnson 1987; Journel 1994). Sequential simulation aims to reproduce spatial properties sequentially by decomposing the multivariate conditional distributions into a set of univariate distributions. Considering a stationary and ergodic random field $\varvec{Z}\left( \varvec{u} \right)$, let $Z\left( {\varvec{u}_{1} } \right), \ldots ,Z\left( {\varvec{u}_{N} } \right)$ be a set of random variables with locations at $\varvec{u}_{1} , \ldots ,\varvec{u}_{N}$, respectively. Then, the $N$ random variables $Z\left( {\varvec{u}_{1} } \right), \ldots ,Z\left( {\varvec{u}_{N} } \right)$ constitute a joint multivariate distribution. In terms of the stochastic simulation, it is supposed that realizations are to be generated from $Z\left( {\varvec{u}_{1} } \right), \ldots ,Z\left( {\varvec{u}_{N} } \right)$, and the available data set is ${{\Lambda }}_{0} = \left\{ {\zeta \left( {\varvec{u}_{1}^{'} } \right), \ldots ,\zeta \left( {\varvec{u}_{n}^{'} } \right)} \right\}$, where $\zeta \left( {\varvec{u}_{i}^{'} } \right)$ is the sample data at the location $\varvec{u}_{i}^{'}$ for $i = 1, \ldots ,n$ and $n$ is the number of sample data in total. For simplification, $Z\left( {\varvec{u}_{1} } \right), \ldots ,Z\left( {\varvec{u}_{N} } \right)$ are alternatively written as $Z_{1} , \ldots ,Z_{N}$, and a similar simplification of notation applies in the context of a random field. Following the above notation, the stochastic simulation of the random field is based on the sampling from the $N$-variate probability distribution posterior to the data set ${\Lambda }_{0}$, which can be characterized by a ccdf as $F_{\varvec{Z}} (z_{1} , \ldots ,z_{N} |{\Lambda }_{0} )$ or by a cpdf as $f_{\varvec{Z}} (z_{1} , \ldots ,z_{N} |{\Lambda }_{0} )$. The joint cpdf $f_{\varvec{Z}} (z_{1} , \ldots ,z_{N} |{\Lambda }_{0} )$. can be decomposed into the product of a series of univariate cpdfs (Rosenblatt 1952; Johnson 1987) as

1 $$ {f_{Z} \left({z_{1}, \ldots,z_{N} |\Lambda_{0}} \right) = f_{{Z_{1}}} \left({z_{1} |\Lambda_{0}} \right) \cdots f_{{Z_{N}}} \left({z_{N} |\Lambda_{N - 1}} \right),} $$

where ${\Lambda }_{i} \left( {i = 1, \ldots ,N - 1} \right)$ is a series of sets and ${\Lambda }_{i} = {\Lambda }_{i - 1} \cup \left\{ {\zeta \left( {\varvec{u}_{i} } \right)} \right\},  i = 1, \ldots ,N$, where $\zeta \left( {\varvec{u}_{i} } \right)$ is the value drawn from the conditional probability distribution with a density function described as $f_{{Z_{i} }} \left( {z_{i} |{\Lambda }_{i - 1} } \right)$.

The basic idea of sequential simulation is to sequentially draw random values from the decomposed univariate cpdfs through a random path that visits all the nodes to be simulated. Irrespective of the node’s location corresponding to the sequence number, there is no difference in the sampling procedures. Without loss of generality, the cpdf in every single sampling procedure can be symbolized uniformly as $f_{{Z_{0} }} (z_{0} |{\Lambda })$, where $Z_{0}$ means the current simulating node and ${\Lambda }$ means the set of conditioning data around $Z_{0}$’s location $\varvec{u}_{0}$. Considering the computational intensity and the statistical relevancy, the conditioning data are usually confined to a neighborhood closest to the simulation node instead of taking account of all available data on the whole domain of the random field. For more details on this screen-effect approximation, the reader is referred to Dimitrakopoulos and Luo (2004).

An algorithmic description of sequential simulation can be summarized as the following steps:

1. Draw a random path to visit all the $N$ nodes to be simulated.
2. Starting from $i = 1$ and for each node $Z\left( {\varvec{u}_{i} } \right)$, derive the conditional probability cumulative distribution $F_{{Z_{i} }} (z_{i} |{\Lambda }_{i - 1} )$ or the density function $f_{{Z_{i} }} (z_{i} |{\Lambda }_{i - 1} )$.
3. Draw a random value $\zeta \left( {\varvec{u}_{i} } \right)$ from the conditional probability distribution in step (2) and update the conditioning data by adding the node value $\zeta \left( {\varvec{u}_{i} } \right)$ into the current data set ${\Lambda }_{i}$.
4. Repeat from step (2) until all the nodes are visited.

### High-Order Spatial Legendre Moments

In probability theory, moments are defined as expectations of integer power functions of a random variable. Given a random variable $Z$ in probability space $\left( \Omega, {\mathcal{F}},\,P \right)$, suppose that the density of probability measure *P* is a continuous function $f_{Z} \left( z \right)$. The moment of order $w$ is defined as

2 $$ {{\text{Mom}}_{Z} (w) = E[{Z^{w}} ] = \int_{\Omega} [{Z(\omega )} ]^{w} dP(\omega ) = \int_{{\mathbb{R}}} z^{w} f_{Z} (z )dz.}  $$

**Fig. 1:**
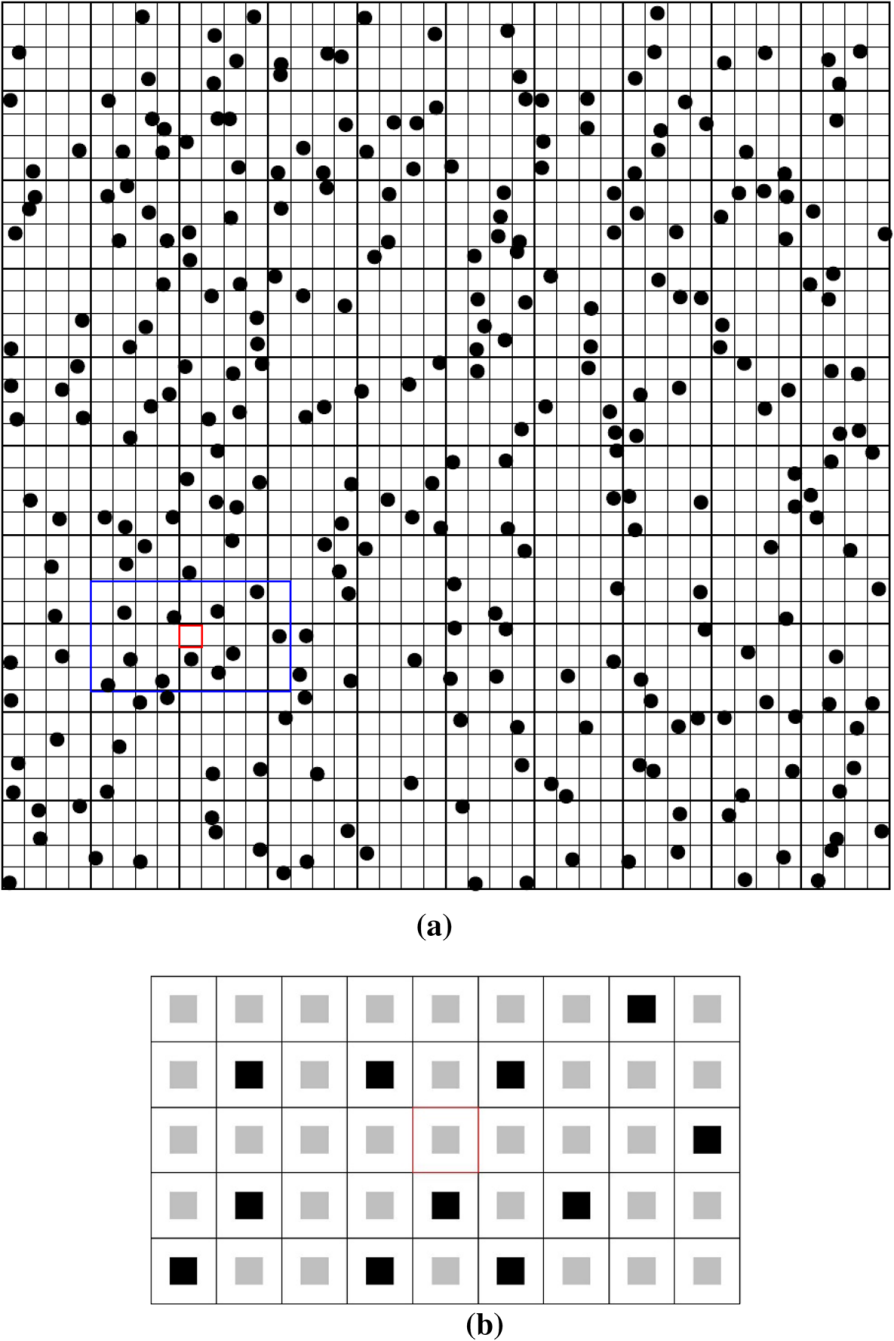
**a** A 40 × 40 grid to be simulated with a 9 × 5 template overlaid on the current visiting node. **b** Spatial template **T** and a certain data event in **T**. The center square is the node to be simulated; the black squares are the conditioning data

The moments of random vector $\varvec{Z} = \left[ {Z_{0} , \ldots ,Z_{N} } \right]$ with a multivariate density $f_{\varvec{Z}} \left( {z_{0} , \ldots ,z_{N} } \right)$ are defined similarly as

3 $$ \begin{array}{*{20}c} {{\text{Mom}}_{\varvec{Z}} \left({w_{0}, \cdots,w_{n}} \right) = E\left[{z_{1}^{{w_{0}}} \cdots z_{N}^{{w_{N}}}} \right] = \displaystyle\int \limits_{{{\mathbb{R}}^{N}  }} z_{0}^{{w_{0}}} \cdots z_{N}^{{w_{N}}} f_{\varvec{Z}} \left({z_{0}, \ldots,z_{N}} \right)dz_{0} \cdots dz_{N},} \\ \end{array} $$

where $w_{i} (i = 0, \cdots ,N$) are the orders of moments for the *i*th element of vector **Z**. The spatial moments of a discrete random field ${\mathbf{Z}} = \left[ {Z\left( {\varvec{u}_{0} } \right), \ldots ,Z\left( {\varvec{u}_{n} } \right)} \right]$ are functions of spatial location variables $\varvec{u}_{0} , \ldots ,\varvec{u}_{n}$. Assuming the random field ${\mathbf{Z}}\left( \varvec{u} \right)$ is stationary and ergodic, the spatial moments of ${\mathbf{Z}}\left( \varvec{u} \right)$ can be expressed as functions of distance vectors, and, thus, they are independent of the locations. These distance vectors, which keep the spatial configuration of a center node and nodes within its neighborhood, can be expressed using a spatial template **T** (Fig. 1). The terminologies of the spatial template **T** and data events (Strebelle 2002; Dimitrakopoulos et al. 2010) are as follows:

(i) Spatial template **T**: geometry defined by *N* distance vectors $\left( {\varvec{h}_{1} , \ldots ,\varvec{h}_{N} } \right)$ from the center node $\varvec{u}_{0}$, ${\mathbf{T}} = \left\{ {\varvec{u}_{0} ,\varvec{u}_{0} + \varvec{h}_{1} , \ldots ,\varvec{u}_{0} + \varvec{h}_{N} } \right\}$.
(ii) Data events: outcomes of the random field in the spatial template **T**. Specifically, the data events are conditioning data set ${\Lambda }$ in the present work.

The spatial moments of a random field **Z** in a template **T** can be expressed element-wise as

4 $$ \begin{array}{*{20}c} {{\text{Mom}}_{{\mathbf{Z}}}^{{\mathbf{T}}} \left({w_{0}, \cdots,w_{N}} \right) = E\left[{\varvec{h}_{1}, \ldots,\varvec{h}_{N};Z_{0}^{{w_{0}}} \cdots Z_{N}^{{w_{N}}}} \right],} \\ \end{array} $$

where ${\text{Mom}}_{{\mathbf{Z}}}^{{\mathbf{T}}} \varvec{ }$ is the moment function of ${\mathbf{Z}}$ in the spatial template **T**, $\left( {\varvec{h}_{1} , \ldots ,\varvec{h}_{N} } \right)$ are the distance vectors to represent the geometry of **T**, and $w_{i}$ are the orders of the moments with each random variable $Z\left( {\varvec{u}_{i} } \right)$($i = 1, \ldots ,N)$.

The Legendre polynomials are used here to further define the concept of spatial Legendre moments. Legendre polynomials are one kind of special math functions defined on the interval [− 1, 1], which can be expressed using Rodrigues’ formula (Zarowski 2004)

5 $$ \begin{array}{*{20}c} {P_{m} \left(z \right) = \frac{1}{{2^{m} m!}}\frac{{d^{m}}}{{dz^{m}}}\left[{\left({z^{2} - 1} \right)^{m}} \right],} \\ \end{array} $$

where $P_{m} \left( z \right)$ is the *m*th-degree Legendre polynomial. The infinite sequence of polynomials forms a complete orthogonal basis set on the domain D = [− 1, 1]. The orthogonal property of the Legendre polynomials can be expressed as

6 $$ \begin{array}{ll} {\displaystyle\int \limits_{D} P_{m} \left(z \right)P_{n} \left(z \right)dz = \left\{{\begin{array}{ll} 0& \quad m \ne n \\ {\frac{2}{2m + 1}}    & \quad {m = n} \\ \end{array}} \right. ,} \\ \end{array} $$

and the norm of the Legendre polynomial $P_{m} \left( z \right)$ is

7 $$ ||P_{m}|| = \sqrt {\frac{2}{2m + 1}}. $$

With a simple substitution of polynomials in moment function Eq. (4) into Legendre polynomials, the spatial Legendre moments are defined as

8 $$ \begin{array}{*{20}c} {L_{{w_{0} w_{1} \cdots w_{N}}}^{\varvec{T}} = \mathop \prod \limits_{i = 0}^{N} \left({w_{i} + \frac{1}{2}} \right) \cdot E\left[{h_{1}, \ldots,h_{N};P_{{w_{0}}} \left({z_{0}} \right)P_{{w_{1}}} \left({z_{1}} \right) \cdots P_{{w_{N}}} \left({z_{N}} \right)} \right],} \\ \end{array} $$

where $L_{{w_{0} w_{1} \cdots w_{N} }}^{\varvec{T}}$ are Legendre moments defined on the spatial template **T**; the extra coefficient $\left( {w_{i} + \frac{1}{2}} \right)$ on the right-hand side of the equation is intentionally introduced as a normalization term for the convenience of the later computation (see the Appendix for details).

### Multivariate Expansion Series of a Joint pdf

A piecewise continuous function $f\left( z \right)$ defined on the interval [− 1, 1] can be written as a series of Legendre polynomials

9 $$ \begin{array}{*{20}c} {f\left(z \right) = \mathop \sum \limits_{m = 0}^{\infty} L_{m} P_{m} \left(z \right).} \\ \end{array} $$

Likewise, the expansion of a multivariate function $f\left( {z_{0} ,z_{1} , \ldots ,z_{N} } \right)$ can be defined on an (*N * + 1)-dimensional domain in the same way. Specifically, suppose that the multivariate function is a density function related to the joint distribution of random variables on a spatial template **T**. The density function can be expanded into Legendre polynomial series in terms of Legendre spatial moments and Legendre polynomials as (see the Appendix for details)

10 $$ \begin{array}{*{20}c} {f\left({z_{0},z_{1}, \ldots,z_{N}} \right) = \mathop \sum \limits_{{w_{0} = 0}}^{\infty} \mathop \sum \limits_{{w_{1} = 0}}^{\infty} \cdots \mathop \sum \limits_{{w_{N} = 0}}^{\infty} L_{{w_{0} w_{1} \cdots w_{N}}}^{\varvec{T}} P_{{w_{0}}} \left({z_{0}} \right)P_{{w_{1}}} \left({z_{1}} \right) \cdots P_{{w_{N}}} \left({z_{N}} \right).} \\ \end{array} $$

In practice, the above infinite series in Eq. (10) is truncated at a certain order W, thus leading to the approximated density function

11 $$ \begin{array}{*{20}c} {f\left({z_{0},z_{1}, \ldots,z_{N}} \right) \approx f_{W} \left({z_{0},z_{1}, \ldots,z_{N}} \right) = \mathop \sum \limits_{{w_{0} = 0}}^{W} \mathop \sum \limits_{{w_{1} = 0}}^{W} \cdots \mathop \sum \limits_{{w_{N} = 0}}^{W} L_{{w_{0} w_{1} \cdots w_{N}}}^{\varvec{T}} \mathop \prod \limits_{i = 0}^{N} P_{{w_{i}}} \left({z_{i}} \right).} \\ \end{array} $$

From the definition in Eq. (8), the spatial Legendre moments can be explicitly derived as

12 $$ \begin{array}{*{20}c} {L_{{w_{0} w_{1} \cdots w_{N}}}^{\varvec{T}} = \displaystyle\int \limits_{D} \mathop \prod \limits_{i = 0}^{N} \left[{\left({w_{i} + \frac{1}{2}} \right)P_{{w_{i}}} \left({z_{i}} \right)} \right]f\left({z_{0},z_{1}, \ldots, z_{N}} \right)dz_{0} dz_{1} \cdots dz_{N}.} \\ \end{array} $$

Experimentally, if there are *M* replicates of data events associated with template **T** found in the TI, the spatial Legendre moments can be calculated as

13 $$ \begin{array}{*{20}c} {\tilde{L}_{{w_{0} w_{1} \cdots w_{N}}}^{\varvec{T}} = \frac{1}{M} \mathop \sum \limits_{t = 1}^{M} \mathop \prod \limits_{i = 0}^{N} \left({w_{i} + \frac{1}{2}} \right)P_{{w_{i}}} \left({\zeta_{t,i}} \right),} \\ \end{array} $$

where $\zeta_{t,i}$ are the data values of replicates in the template **T**, $t$ is the sequence number of replicates, and $i$ is the sequence number of random variables.

## Computational Model

Combining Eqs. (10)–(13), the empirical joint pdf can be derived as

14 $$ \begin{array}{*{20}c} {\begin{array}{*{20}c} {\tilde{f}\left({z_{0},z_{1}, \ldots, z_{N}} \right) \approx \tilde{f}_{W} \left({z_{0},z_{1}, \ldots, z_{N}} \right)} \\ {\qquad \qquad = \frac{1}{M} \mathop \sum \limits_{t = 1}^{M} \mathop \sum \limits_{{w_{0} = 0}}^{W} \mathop \sum \limits_{{w_{1} = 0}}^{W} \cdots \mathop \prod \limits_{i = 0}^{N} \left[{\left({w_{i} + \frac{1}{2}} \right)P_{{w_{i}}} \left({\zeta_{t,i}} \right)P_{{w_{i}}} \left({z_{i}} \right)} \right]} \\ {= \frac{1}{M} \mathop \sum \limits_{t = 1}^{M} \mathop \prod \limits_{i = 0}^{N} \left[{\mathop \sum \limits_{w = 0}^{W} \left({w + \frac{1}{2}} \right)P_{w} \left({\zeta_{t,i}} \right)P_{w} \left({z_{i}} \right)} \right]} \\ \end{array}.} \\ \end{array} $$

Equation (14) gives a unified computational model of empirical estimation of the density function on the spatial template **T**, noticing that, on the right-hand side of the equation, the subscript $i$ of $w_{i}$ is dropped because of the symmetry of computation.

Now let’s consider the cpdf $f_{{Z_{0} }} \left( {z_{0} | {\Lambda }} \right)$ of a single sampling step in sequential simulation (ref. Sect. 2.1). The joint pdf can be marginalized from Eq. (14) to get the marginal pdf of conditioning random variables. To specify the difference between the empirical models and theoretical models in Eqs. (10) and (11), $\tilde{f}$ and $\tilde{f}_{W}$ specifically denote the experimental function corresponding to pdf $f$ and its Legendre polynomial series truncated at order $W$, respectively.

For convenience, denote functions $X_{t} \left( {z_{i} } \right)$ as

15 $$ \begin{array}{*{20}c} {X_{t} \left({z_{i}} \right) = \mathop \sum \limits_{w = 0}^{W} \left({w + \frac{1}{2}} \right)P_{w} \left({\zeta_{t,i}} \right)P_{w} \left({z_{i}} \right).} \\ \end{array} $$

Then, Eq. (14) can be rewritten as

16 $$ \begin{array}{*{20}c} {\tilde{f}_{W} \left({z_{0},z_{1}, \ldots, z_{N}} \right) = \frac{1}{M}\mathop \sum \limits_{t = 1}^{M} X_{t} \left({z_{0}} \right)\mathop \prod \limits_{i = 1}^{N} X_{t} \left({z_{i}} \right).} \\ \end{array} $$

The result of the integration of $X_{t} \left( z \right)$ over [− 1, 1] can be derived from the orthogonal properties of Legendre polynomials as

17 $$ { \int _{- 1}^{1} X_{t} \left({z_{i}} \right)dz_{i} = 1.} $$

In fact, Eqs. (16) and (17) ensure that the integral of the approximated pdf to be 1, a necessary property of probability density.

Followed by the marginalization and Eq. (17), the empirical density of marginal distribution on the random variables $z_{1} , \ldots ,z_{N}$ is

18 $$ \begin{array}{*{20}c} {\tilde{f}_{W} \left({z_{1}, \ldots, z_{N}} \right) = \frac{1}{M}\mathop \sum \limits_{t = 1}^{M} \mathop \prod \limits_{i = 1}^{N} X_{t} \left({z_{i}} \right).} \\ \end{array} $$

From Eqs. (16) and (18) and considering the relation between the cpdf and the joint pdf, one can derive

19 $$  {f\left({z_{0} | \Lambda} \right) \approx \tilde{f}_{W} \left({z_{0} | \Lambda} \right) = \frac{{\mathop \sum \nolimits_{t = 1}^{M} X_{t} \left({z_{0}} \right) \cdot \mathop \prod \nolimits_{i = 1}^{N} X_{t} \left({\zeta_{i}} \right)}}{{\mathop \sum \nolimits_{t = 1}^{M} \mathop \prod \nolimits_{i = 1}^{N} X_{t} \left({\zeta_{i}} \right)}},} $$

which provides a concise computational model of the cpdf.

The above development provides a theoretical equivalency of the approximation of the cpdf by a truncated Legendre series, which was proposed by Mustapha and Dimitrakopoulos (2010b, 2011). However, the new reformulated model in the current paper leads to a different stochastic simulation method in view of the related computational aspects. The advantage of the new model represented by Eq. (19) is that no explicit computations of moments or cumulants are needed. In addition, the new model is computationally more accurate than the hosim program in Mustapha and Dimitrakopoulos (2011), in which some terms have to be dropped from the full expansion of the Legendre series in the form of spatial cumulants to gain computational efficiency.

## Algorithm Description and Computational Analysis

### Algorithm for Computing a cpdf

From Eqs. (17)–(19), it can be easily shown that

20 $$  {\int_{- 1}^{1} \tilde{f}_{W} \left({z_{0} | \Lambda} \right)dz_{0} = 1.}  $$

As $X_{t} \left( {\zeta_{t,i} } \right)$ is a constant from Eq. (15) and from Eqs. (15) and (19), it is obvious that $\tilde{f}_{W} \left( {z_{0} | {\Lambda }} \right)$ can be expressed as the summation of a series of Legendre polynomials, that is

21 $$ \begin{array}{*{20}c} {\tilde{f}_{W} \left({z_{0} | \Lambda} \right) = \mathop \sum \limits_{w = 0}^{W} c_{w} P_{w} \left({z_{0}} \right),} \\ \end{array} $$

where $c_{w} \left( {w = 1, \ldots ,W} \right)$ are constants which can be conveniently computed as shown in the following Algorithm 1.

By the property of Legendre polynomials that $P_{0} \left( z \right) = 1,\forall z \in \left[ { - 1,1} \right]$, combined with Eqs. (15) and (21), the computation of coefficients $c_{w} \left( {w = 1, \ldots ,W} \right)$ can be divided into the computation of functions $X_{t} \left( {z_{i} } \right)$ over the nodes of each replicate. Especially, the first term of $c_{w}$ is always fixed as $c_{0} = \frac{1}{2}$.


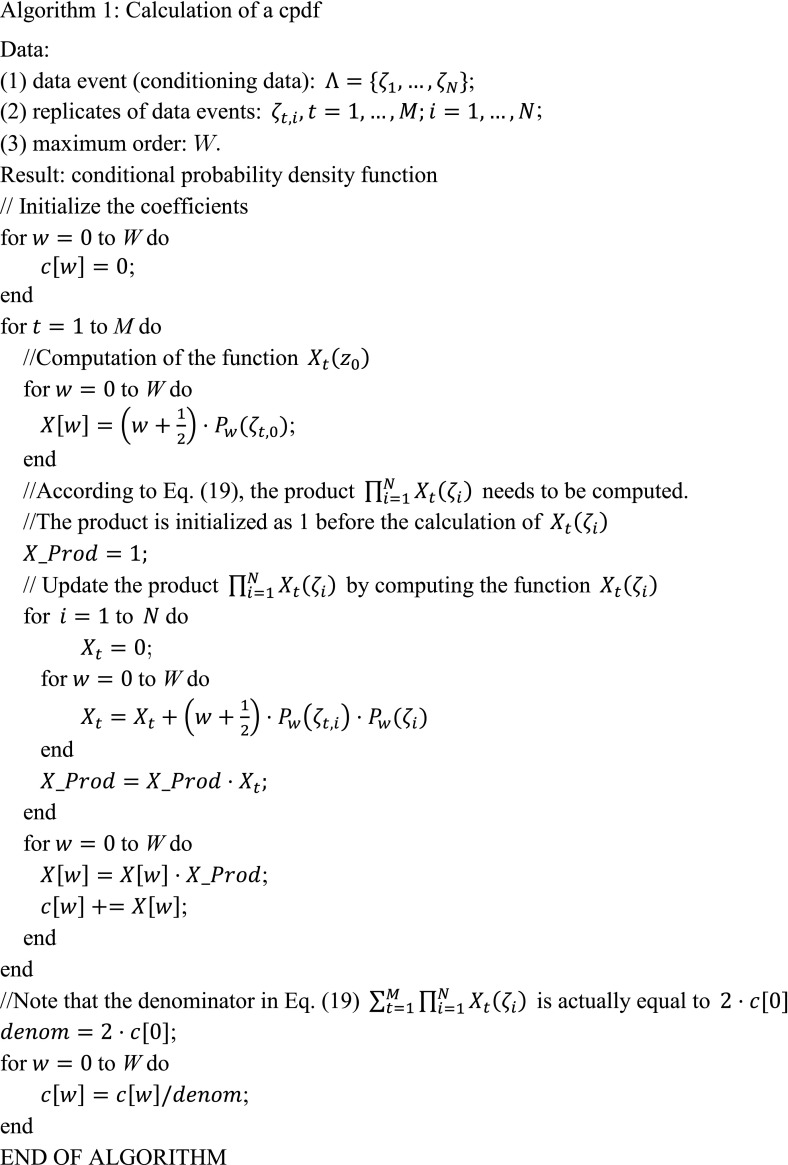


### Recursive Algorithm for Computing a ccdf

From the results of Algorithm 1, the cpdf can be expressed as

22 $$ \begin{array}{*{20}c} {f\left({z_{0} | \Lambda} \right) = \frac{1}{2} + \mathop \sum \limits_{w = 1}^{W} c_{w} P_{w} \left({z_{0}} \right).} \\ \end{array} $$

The coefficient $c_{0} = \frac{1}{2}$ is taken out from the summation in Eq. (22) so that the Bonnet’s recursion relation of Legendre polynomials can be smoothly applied in the followed derivation.

According to the Bonnet’s recursion relation of Legendre polynomials

23 $$ \begin{array}{*{20}c} {\left({2w + 1} \right)P_{w} \left(z \right) = \frac{d}{dz}\left[{P_{w + 1} \left(z \right) - P_{w - 1} \left(z \right)} \right],} \\ \end{array} $$

the following equation can be derived

24 $$ \begin{array}{*{20}c} {\left({2w + 1} \right)\int _{- 1}^{{z_{0}}} P_{w} \left(z \right)dz = P_{w + 1} \left({z_{0}} \right) - P_{w - 1} \left({z_{0}} \right)} \\ \end{array} .$$

Therefore, the ccdf, $F\left( {z_{0} | {\Lambda }} \right)$, can be deduced as

25 $$ \begin{array}{*{20}c} {\begin{array}{*{20}c} {F\left({z_{0} | \Lambda} \right) = \int _{- 1}^{{z_{0}}} f\left({z_{0} | \Lambda} \right)dz} \\ \quad{= \frac{1}{2} + \frac{1}{2}z_{0} + \mathop \sum \limits_{w = 1}^{W} \frac{{c_{w}}}{2w + 1}\left[{P_{w + 1} \left({z_{0}} \right) - P_{w - 1} \left({z_{0}} \right)} \right]} \\ {= \mathop \sum \limits_{w = 0}^{W + 1} d_{w} P_{w} \left({z_{0}} \right)} \\ \end{array}.} \\ \end{array} $$

As can be seen from Eq. (25), the ccdf is also expressed as the summation of the univariate Legendre polynomials, with the order of the Legendre polynomials increasing by one because of the integration. Furthermore, the new coefficients $d_{w} \left( {w = 0, \ldots ,W,W + 1} \right)$ can now be computed through Eq. (25) in an iterative way, as shown in Algorithm 2.


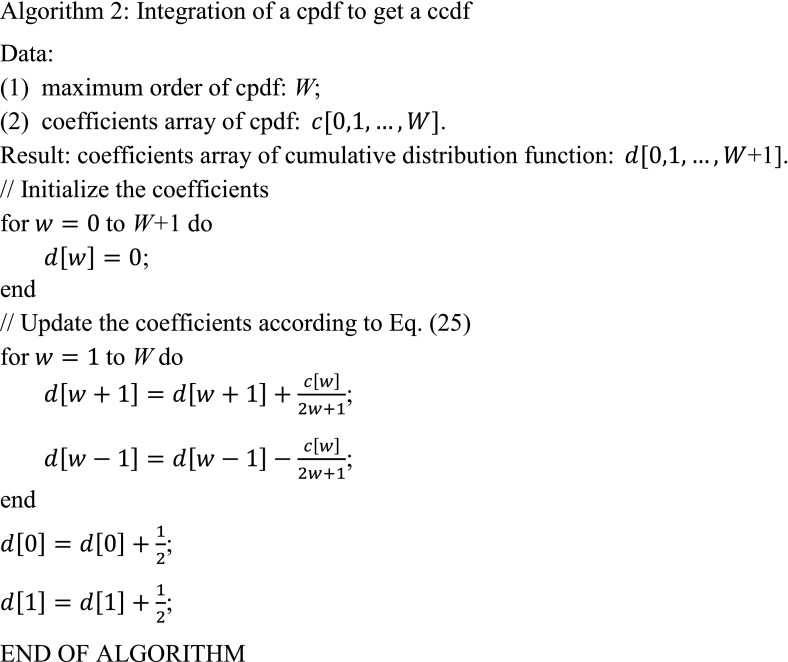


### Computational Complexity

The most computationally demanding part of the high-order simulation algorithm is to calculate the Legendre series coefficients, which is the basis for estimating the cpdfs. Considering that the cpdfs are approximated by Legendre series truncated to a certain order *W*, as Eq. (11) shows, the number of the different coefficients is $\left( {W + 1} \right)^{N + 1}$, where *N* is the number of data points. Even the Legendre series is approximated by truncated series, where the sum of orders of different variables is not greater than *W*, which is the form adopted by Mustapha and Dimitrakopoulos (2011). The number of the different coefficients is still as big as $$ \mathop \sum \limits_{w = 0}^{W} \left( {\begin{array}{*{20}c} {N + w} \\ w \\ \end{array} } \right) $$ for a single data event. Although this computational complexity can be reduced by discarding some terms which are regarded as negligible, it should be noted that this simplification may lead to a loss of accuracy.

From Eqs. (15) and (19), it can be seen that all of the different coefficients introduced by the explicit expansion of Legendre series are reduced to a calculation of the function $\mathop \prod \nolimits_{i = 1}^{N} X_{t} \left( {z_{i} } \right)$. There are only $NW$ computations of Legendre polynomials and a few products and additions included in the calculation of the function $\mathop \prod \nolimits_{i = 1}^{N} X_{t} \left( {\zeta_{t,i} } \right)$ for each replicate of the data event encountered in the TI. It should be noted that the computational time still depends on the number of the replicates encountered in the TI, as well as the maximal order of Legendre polynomials and the number of conditionings in the neighborhood. However, the computational cost regarding the above-mentioned parameters is significantly reduced, as opposed to computing the large number of coefficients in the previous version of high-order simulation.

## Implementation

The implementation is relatively straightforward in terms of the above algorithms estimating the cpdf and ccdf according to the framework of sequential simulation. However, a method is proposed in this section to deal with the replicates, aiming to reduce the conflicts of spatial statistics between the sample data and the TI. The main idea of the method is to deliberately select replicates which are similar to the conditioning data within a certain range according to some measure of similarity. The reason for this is that the conditional probability distribution is a one-dimensional intercept from the multivariate joint probability distribution and, therefore, the replicates that are close to the conditioning data are more relevant to estimate this one-dimensional local probability distribution.

**Fig. 2:**
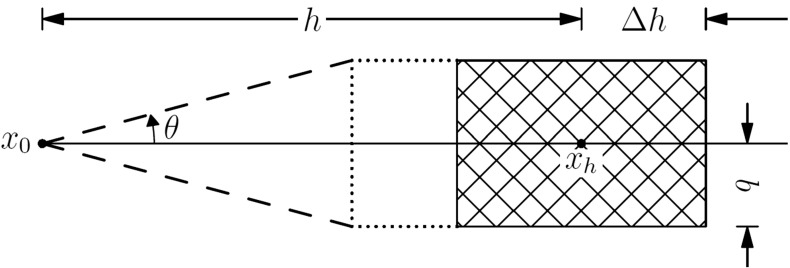
Finding approximate replicates from the training image (TI) with the tolerances of the original geometry template

For every node to be simulated in sequential simulation, a local neighborhood is defined to search for conditional data from both the sample data and the simulation grid. The locations of these conditional data together with the center node to be simulated constitute a geometry template. Given a TI, replicates of the geometry template can always be found from the TI as long as the searching neighborhood is inside of the TI’s extent. In the present work, the measure of similarity between the replicates and the data event is set to be the average square Euclidean distance between the replicates and the conditioning data, and the threshold is set as the variance of the sample data. The replicate will be selected in the estimation of a cpdf if the distance between the replicate and the conditioning data is less than the variance of the sample data. In addition, when there are few replicates that can be found from the TI due to the conflicts between the sample and the TI, some tolerances are given to the shape of the geometry template so that similar replicates can be found. Figure 2 shows a general way to search the candidate points associated with a certain vector in a spatial template. The parameter $\theta$ is the angle tolerance of the candidate point’s deviation from the original vector in the template, and ${\Delta}h$ and $b$ are the tolerances in the lag and bandwidth, respectively. Possible candidate points are taken from the shadowed area, and the point that has the closest property to the ending node of the original vector in the template is selected. To maintain the consistency of the geometry configuration, an inner part of the template is specified such that the relative locations to the center node inside the inner part remains unchanged. In other words, only the nodes further away from the center node are allowed to have the ability to change locations. This strategy gives more flexibility to manipulate the geometry configuration of the replicates.

The main procedure of the high-order simulation approach can be summarized in the following steps

1. Read the sample data and TI into memory. In order to apply the multivariate expansion of Legendre polynomials, the property values of the samples or TI are scaled to the interval [− 1, 1] through a linear transformation.
2. Specify dimensions of a certain neighborhood for searching the conditional data and other parameters, such as the minimum or maximum number of the conditional data. The geometry of the local template totally depends on the locations of the conditional data. In the present work, a rectangular shape neighborhood was used and a searching policy was applied to find the closest points to the center. Nevertheless, the shape of the neighborhood and the searching policy can be manipulated to further control the spatial configuration of the template.
3. Set the lag tolerance, angle tolerance, and bandwidth tolerance to enable searching approximate replicates from the TI (see Fig. 2).
4. Generate a random sequence on the indices of the simulation grid to create a random visiting path.
5. According to the predefined visiting path, sequentially pick one node at a time for the simulation. If the property value is already known (copied from the hard data), then continue to choose another single node until the property value is not assigned. The conditioning data are searched inside the neighborhood centered on the chosen node by the previously specified searching policy from both the hard data and the simulated nodes.
6. A local spatial template is determined by the data and the center node for later simulation. This spatial template is then used to find similar replicates from the TI according to the parameters set in steps (2) and (3). If the number of approximated replicates is not adequate for statistical inference, then drop the furthest node to the center node and repeat until the minimum number of conditioning data is reached.
7. The local ccdf is estimated from the replicates using the algorithms elaborated in Sect. 3. A random value is drawn from the local ccdf using the Monte Carlo method and set as the property value of the node to be simulated.
8. Repeat from step (5) until all the nodes in the random path are visited.

## Examples and Comparisons

**Fig. 3:**
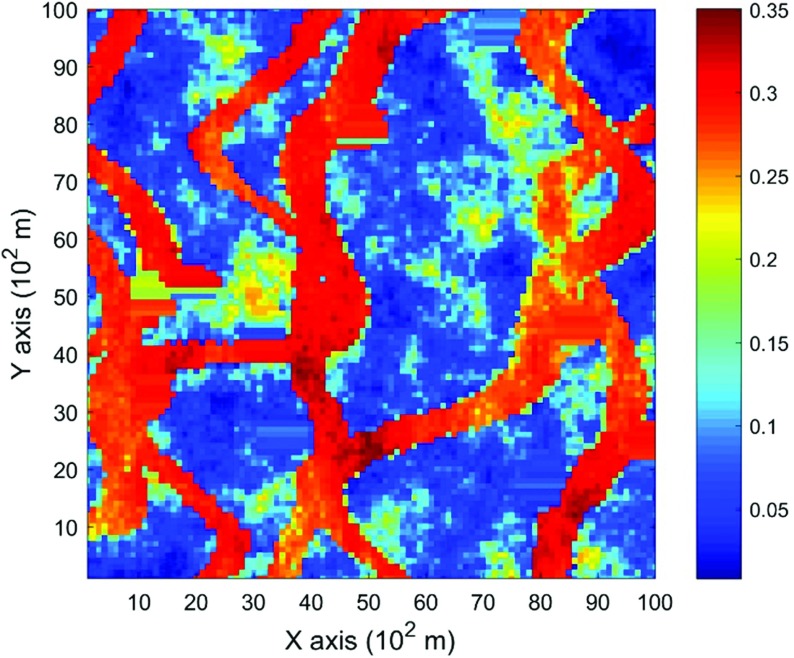
A horizontal section from reservoir’s porosity values with sinuous connectivity

**Fig. 4:**
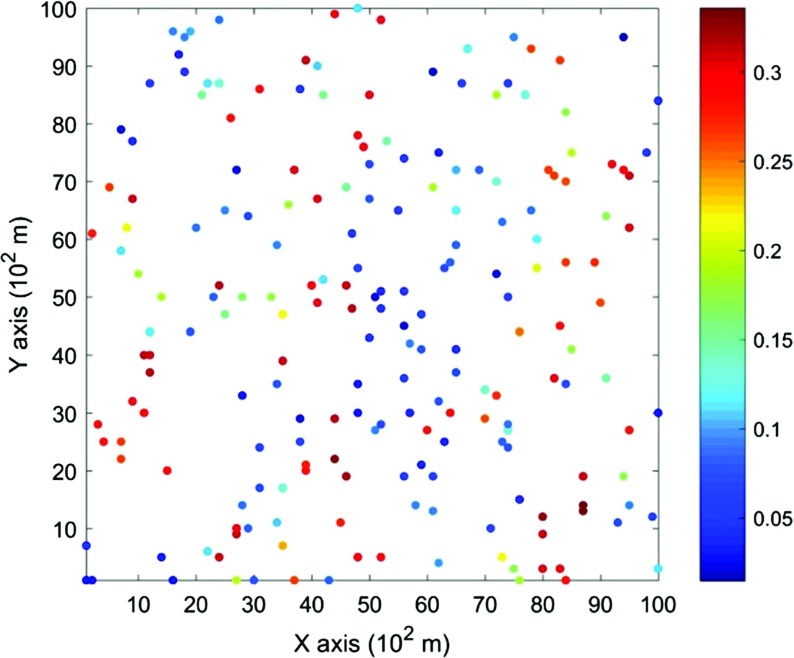
Data points sampled from the exhaustive image (containing 200 points, or 2% of the total data)

**Fig. 5:**
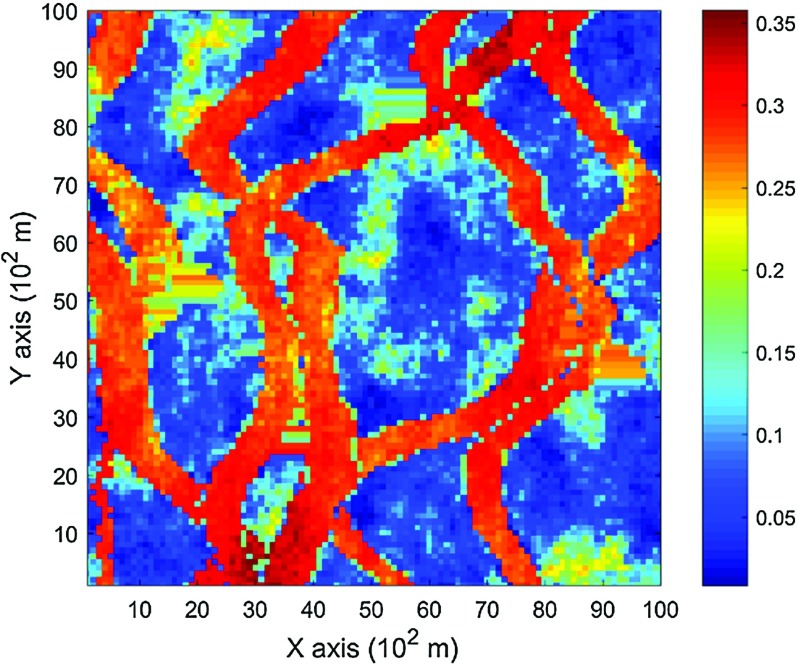
TI that is different from the exhaustive data

The data used in this paper are extracted from the Stanford V reservoir data set (Mao and Journel 1999). A horizontal section serving as the exhaustive image is taken from the Stanford V reservoir model of porosity in a square grid with 100 × 100 pixels (cells of size 100 m). As seen from the exhausting image in Fig. 3, porosity values are distributed as several channels that can be distinguished from the background. For the examples and comparisons presented in the next sections, 200 data points are randomly sampled from the selected exhaustive image to serve as the sample data set and are displayed in Fig. 4. Applying the proposed high-order approach, the selected data are used to simulate the exhaustive image in two different ways, so as to show the sensitivity of the approach to the chosen TI. Accordingly, in Example 1, the exhaustive data are used as the TI; then, in Example 2, the TI is selected from a different section of the Stanford V reservoir data set than the exhaustive image. The second TI is shown in Fig. 5 and has different spatial patterns than those in the exhaustive image. In addition, a comparison of the proposed algorithm to the well-established mps method filtersim (Zhang et al. 2006) is presented. In each of the realizations using the high-order simulation algorithm, a window of size $15 \times 20$ in terms of cell size is used as the search template. The tolerance angle for searching is set to 15°, the lag tolerance to 2 and the bandwidth to 1. These parameters are chosen from the calculation of experimental variograms (Goovaerts 1997). The minimum number of conditioning data is 6 and the maximum number is 12, while 6–12 previously simulated values are used. The maximum order of Legendre polynomials is set to 10. For the realizations generated with filtersim, the searching template is $15 \times 21$ with an inner patch of size $7 \times 7$ and a multiple grid level of 3, while replicates are classified into different categories according to their filter scores. For further details on filtersim, the reader is referred to Zhang et al. (2006).

### Example 1

**Fig. 6:**
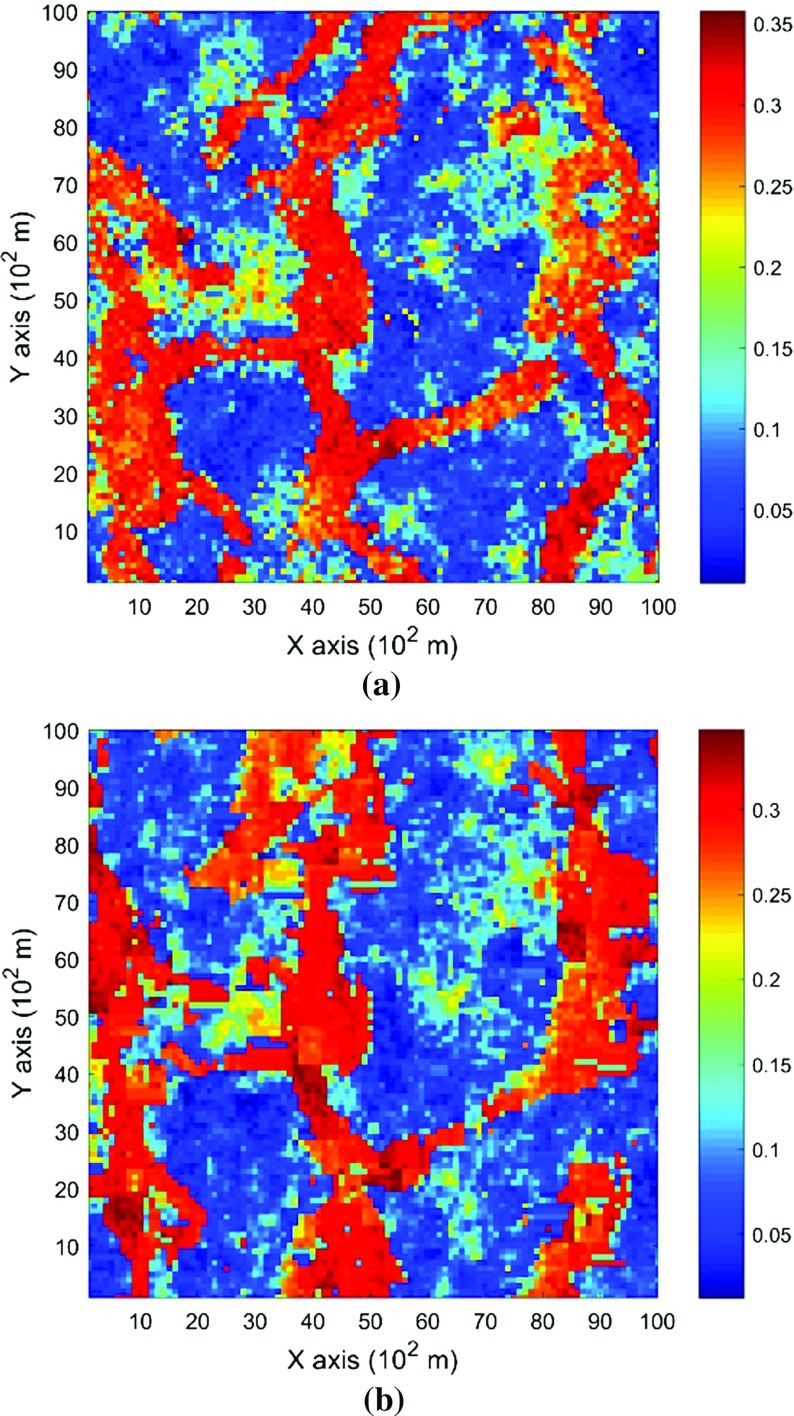
Simulations with 200 sample data using the exhaustive data as the TI: **a** and **b** are one realization from high-order simulation and filtersim, respectively

**Fig. 7:**
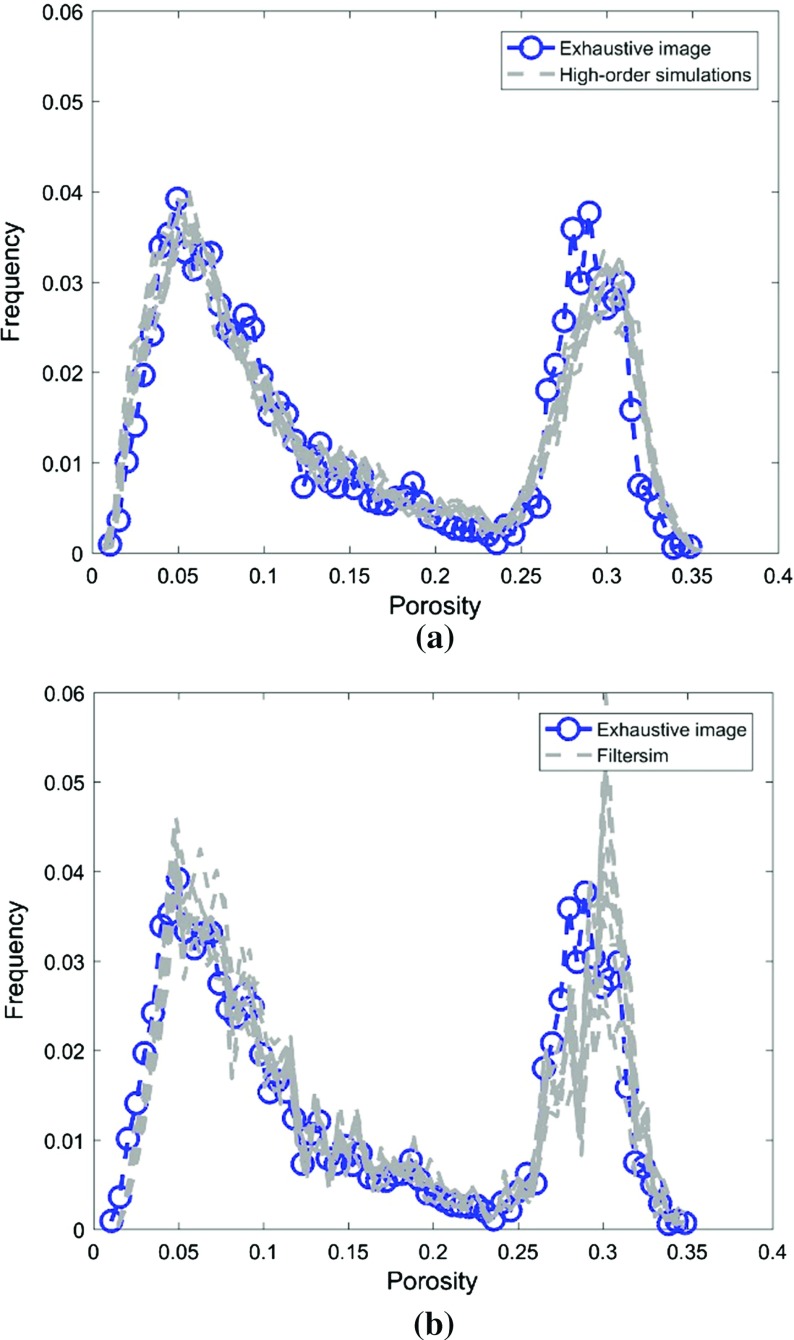
Reproduction of histograms of ten realizations with 200 sample data using the exhaustive data as the TI: **a** and **b** correspond to ten realizations from the high-order simulation and filtersim, respectively

**Fig. 8:**
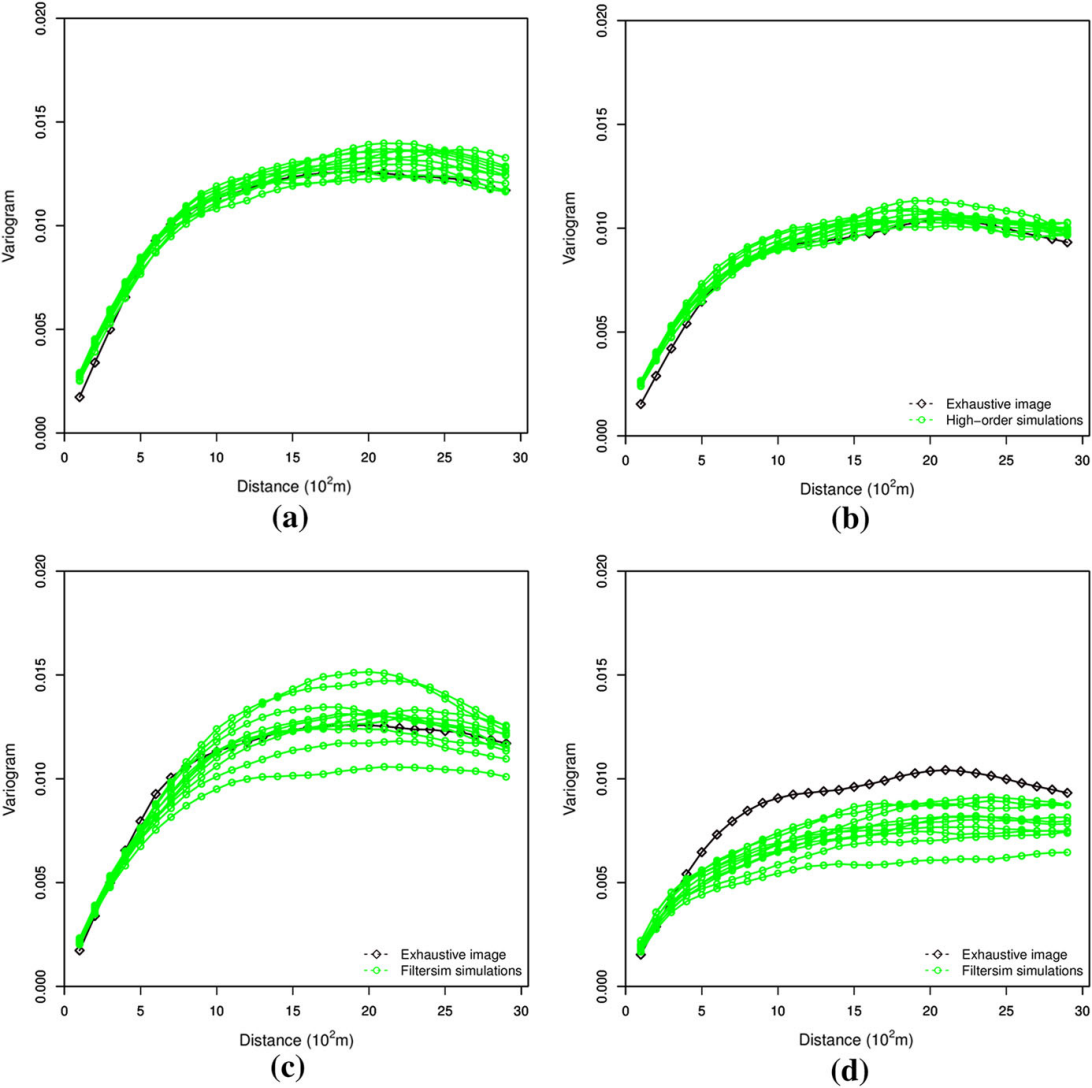
Reproduction of the variograms of ten realizations with 200 sample data using the exhaustive data as the TI from high-order simulation and filtersim, respectively. **a** Reproduction of variograms of high-order simulations in the X-direction. **b** Reproduction of variograms of high-order simulations in the Y-direction. **c** Reproduction of variograms of filtersim simulations in the X-direction. **d** Reproduction of variograms of filtersim simulations in the Y-direction

**Fig. 9:**
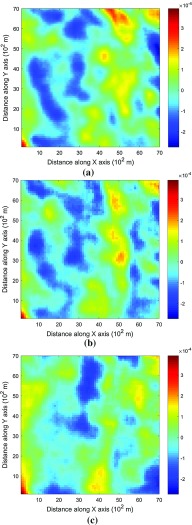
Comparing third-order cumulant maps of realizations with 200 sample data using the exhaustive data as the TI from the high-order simulation and filtersim, respectively. **a** Third-order cumulant map of the exhaustive image. **b** Third-order cumulant map of one realization from the high-order simulation. **c** Third-order cumulant map of one realization from filtersim

This example generates simulations using the 200 samples shown in Fig. 4 and the exhaustive image in Fig. 3 as the TI. In this case, there are no conflicts between the available data and the TI. Figure 6 shows one realization from the high-order simulation and another from filtersim, respectively. From visual comparison with the exhaustive image, the realization from the high-order simulation better reproduces the channels of the original image. To demonstrate the reproduction of the distribution and second-order spatial statistics of simulation results, ten different realizations for each method are generated. The histograms of the realizations are displayed in the Fig. 7 and related variograms are displayed in Fig. 8. Both simulation methods reproduce well the bimodal shape in the histograms; however, in general, high-order simulations show better reproduction in the proportions of porosity values. High-order simulation methods also reproduce well the variograms in the X-direction or Y-direction, while the variograms from the filtersim simulations demonstrate larger fluctuations and have notable deviations from the variogram of the exhaustive data in the Y-direction. For a comparison of the high-order spatial statistics of simulation results to the original data in the two different settings, the third-order cumulant maps are generated by the HOSC program (Mustapha and Dimitrakopoulos 2010c), which are displayed in Fig. 9. This program uses a template with two directions in X-axis and Y-axis, and the number of lags is 70, with lag size as 1. In comparison to the third-order cumulant map of the exhaustive image, the high-order simulation performs better in the reproduction of the high-order statistics, although both simulation methods have reasonable similarity in terms of the third-order cumulant map, as there are no conflicts between the sample data and the TI in this case.

### Example 2

**Fig. 10:**
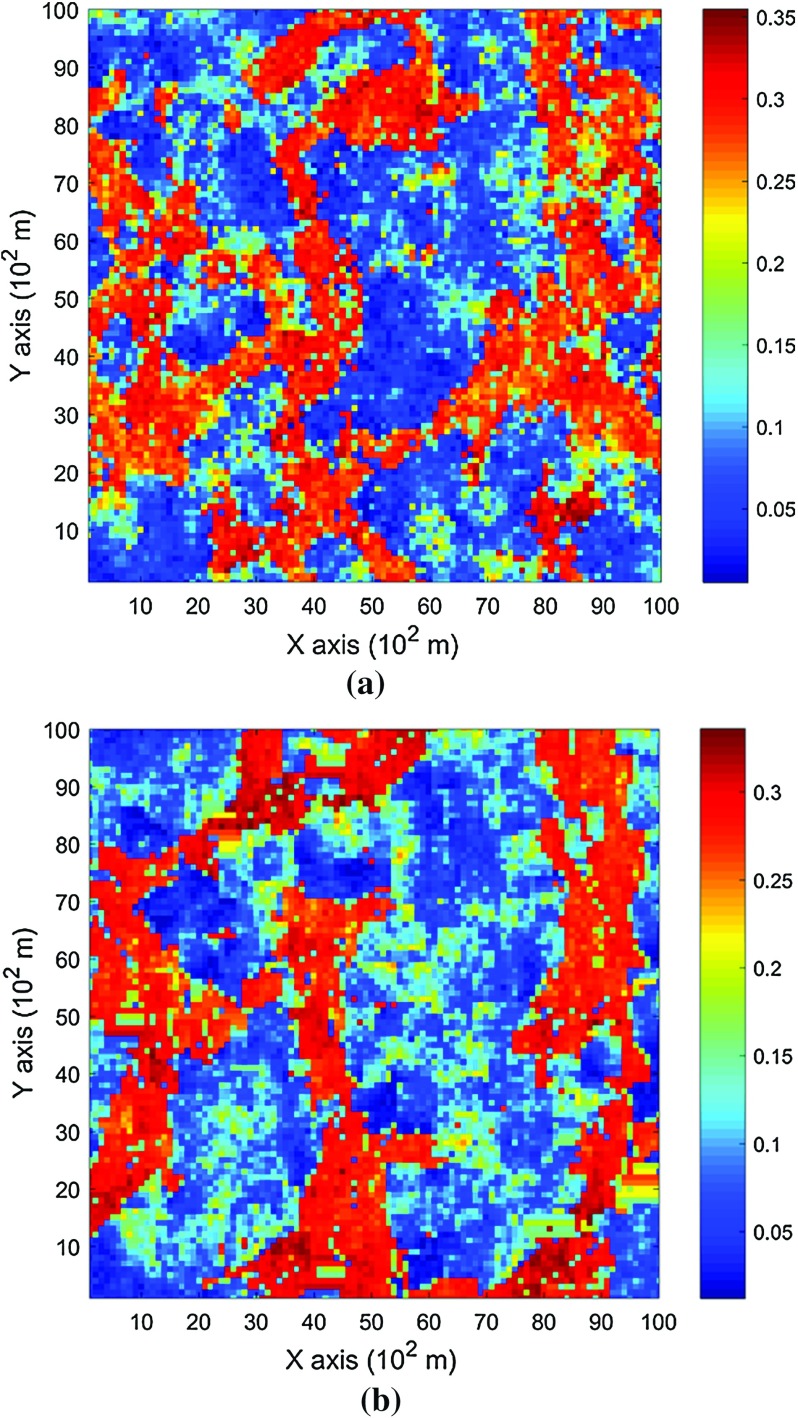
Simulations with 200 sample data using a separate TI different from the exhaustive data: **a** and **b** are one realization from high-order simulation and filtersim, respectively

**Fig. 11:**
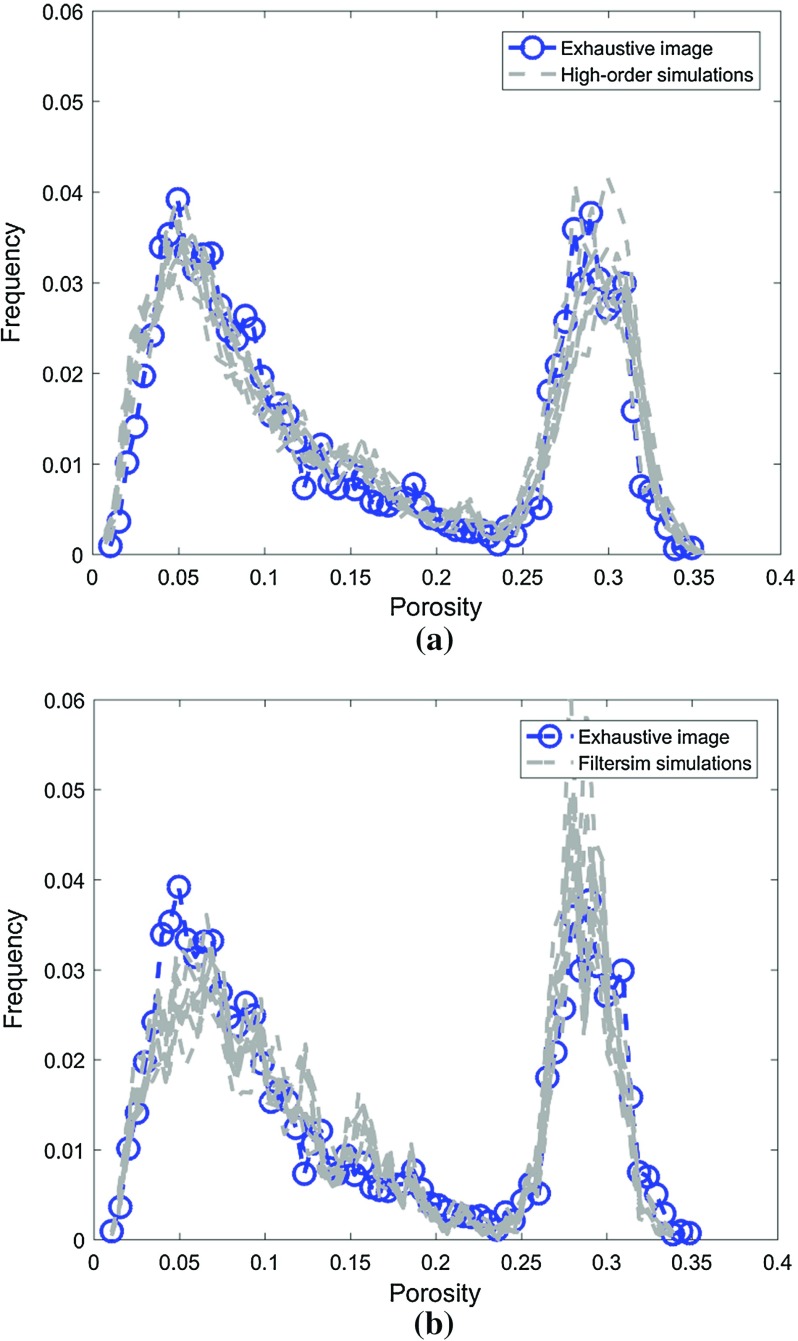
Reproduction of histograms of ten realizations with 200 sample data using the TI different from the exhaustive data: **a** and **b** correspond to ten realizations from high-order simulation and filtersim, respectively

**Fig. 12:**
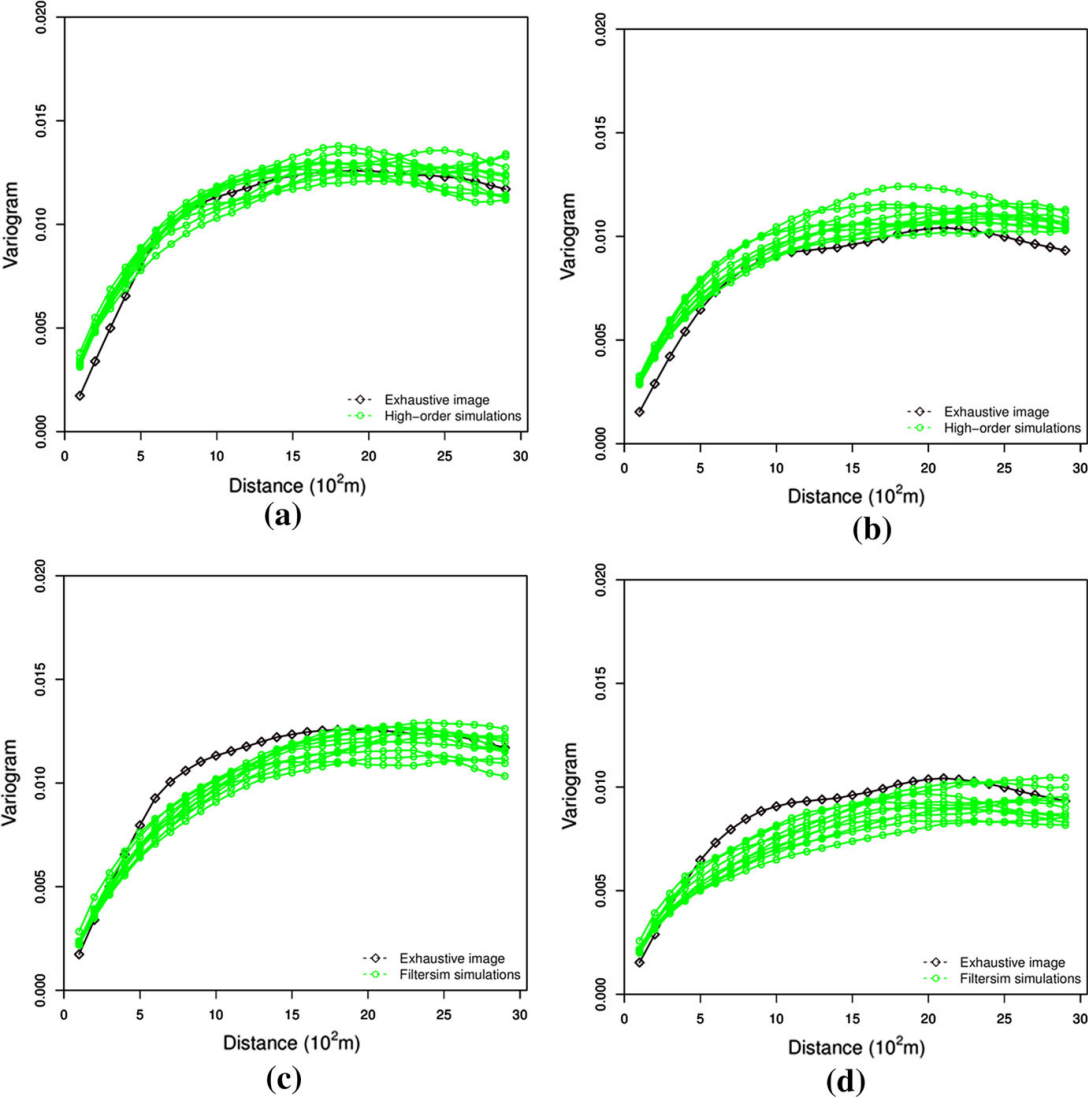
Variograms of ten realizations with 200 sample data using the TI different from the exhaustive data from the high-order simulation and filtersim, respectively

In this setting, the simulations are conducted with the same conditioning data; however, the TI is different from the exhaustive data. Figure 10 shows one realization from the high-order simulation and one for filtersim. Clearly, there are conflicts between the spatial statistics of the sample data and the TI, which are key factors affecting the results of the simulations. As expected, the reproduction of the spatial patterns is worse when compared to the results from the simulations in the previous example. Nevertheless, the realization from the high-order simulation method still maintains the spatial structures of the original exhaustive data. As shown in Fig. 11, the ten realizations of the high-order simulation match the histogram of the exhaustive image very well. By contrast, the ten realizations of filtersim mismatched the exhaustive image in some part of the proportions. From the comparison shown in Fig. 12, the high-order simulation performs better than filtersim in reproducing the variograms of the exhaustive image as well, although there is a minor deviation in the Y-direction.

**Fig. 13:**
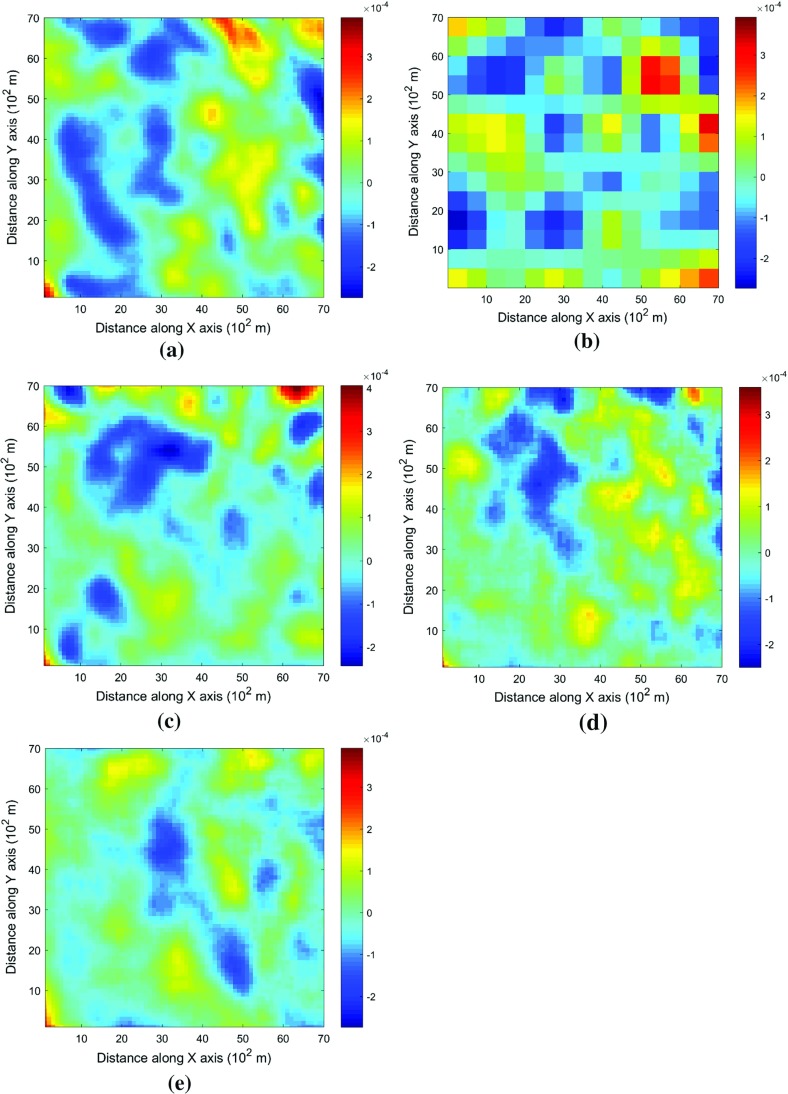
Comparing third-order cumulant maps of realizations with 200 sample data using the TI different from the exhaustive data from the high-order simulation and filtersim, respectively. **a** Third-order cumulant map of the exhaustive image. **b** Third-order cumulant map of the sample data (after smoothing). **c** Third-order cumulant map of the TI. **d** Third-order cumulant map of the realization of high-order simulation. **e** Third-order cumulant map of the realization of filtersim

In order to demonstrate the impact of the conflicts between the sample data and TI during the simulations, Fig. 13 shows the third-order cumulant maps corresponding to the exhaustive image, the sample data, the TI and one realization of high-order simulation and filtersim. The parameter settings to generate the cumulant maps for the grid data are the same as those used in Fig. 9, whereas the lag size is set to 5 grid cells, with the lag tolerance being set to 1 grid cell and the angle tolerance being set to 15° for generating the cumulant map of the sample data. As the sample data is too sparse to compute the cumulant map at the same scale as the exhaustive image, some smoothing has been applied to the cumulant map of the sample data for the purpose of visualization. The third-order cumulant map of the realization from the high-order simulation maintains the main structures of the exhaustive data. On the other hand, the third-order cumulant map of the realization from filtersim resembles the cumulant map of the TI, which differs from the cumulant map of the exhaustive image. This implies that the high-order simulation is primarily data-driven, whereas the filtersim method is TI-driven. This result can be explained by the fact that the high-order simulation seeks to find replicates that comply to the statistical configuration of the conditioning data from the TI, and the values of nodes to be simulated are drawn from the related local probability distribution. By contrast, the filtersim method is TI-driven, which means that the values of nodes to be simulated comes directly from the pasting of certain replicates from the TI, which is patch-based instead of node-by-node, as in the high-order simulation. In particular, the impact of the conditioning data is more important for capturing the large-scale spatial structures in the early stage of the high-order simulation. For instance, Fig. 13b shows the cumulant map of the sample data, and the resolution of the map is much coarser than the exhaustive data. This map shows some distortion when representing the third-order statistics of the exhaustive image due to the sparsity of the data. However, the spatial structures of the limited sample data control the spatial statistics of the results from the high-order simulation. In general, the results in this case study show that the proposed high-order simulation algorithm can reasonably reproduce the overall probability distribution, the second-order statistics, and the higher-order statistical features (such as spatial cumulants), as the statistical conflicts between the sample data and the TI are not severe.

### Parameter Sensitivity Testing

Most parameters in the current implementation of the high-order stochastic simulation method are experimental choices. Amongst all the parameters encountered in the current implementation, some follow common practices in the parameter selection for conventional geostatistical simulations, such as the size of the search window, the lag, and angle tolerance. Additionally, in the high-order simulation method presented here, the number of conditioning data corresponding to a certain template needs more consideration, as it determines the dimension of the local probability distribution. In the current implementation, the number of the conditioning data is limited for two important reasons. First, the limited number of conditioning data reduces the computational time needed to estimate the cpdf. Second, the method resembles the so-called multiple grid strategy (Strebelle 2002) applied in many multi-point simulation methods in order to maintain both large- and small-scale spatial structures. In the early stage of the simulation process, the neighborhoods are more likely to capture large-scale patterns, since the known data are sparse. The neighborhoods gradually correspond to finer-scale patterns as the simulation continues and more known data are generated. A similar search strategy has also been applied and discussed by Mariethoz et al. (2010).

The maximum order of the polynomials is another parameter of importance in the high-order simulation, since it affects the precision of the approximation of a cpdf by a truncated Legendre polynomial series. Theoretically, the coefficients in the Legendre polynomial series decay exponentially, and, in general, much faster than in Taylor series (Cohen and Tan 2012; Wang and Xiang 2012). The numerical results of Cohen and Tan (2012) show that Legendre polynomial series with six non-zero coefficients (orders 10 and 11 in their examples) are highly accurate approximations to the targets. The numerical test to approximate a probability distribution regarding the order of Legendre polynomial series has also been investigated by Mustapha and Dimitrakopoulos (2010b) and led to similar results. However, it should be noted that the above tests are conducted for the approximation of a determined function, whereas for the approximation of the pdf, there is also the impact from the limitation of the number of replicates. Depending on different data sets, Legendre polynomial series with an order from 6 to 20 should be a reasonable range to select.

**Fig. 14:**
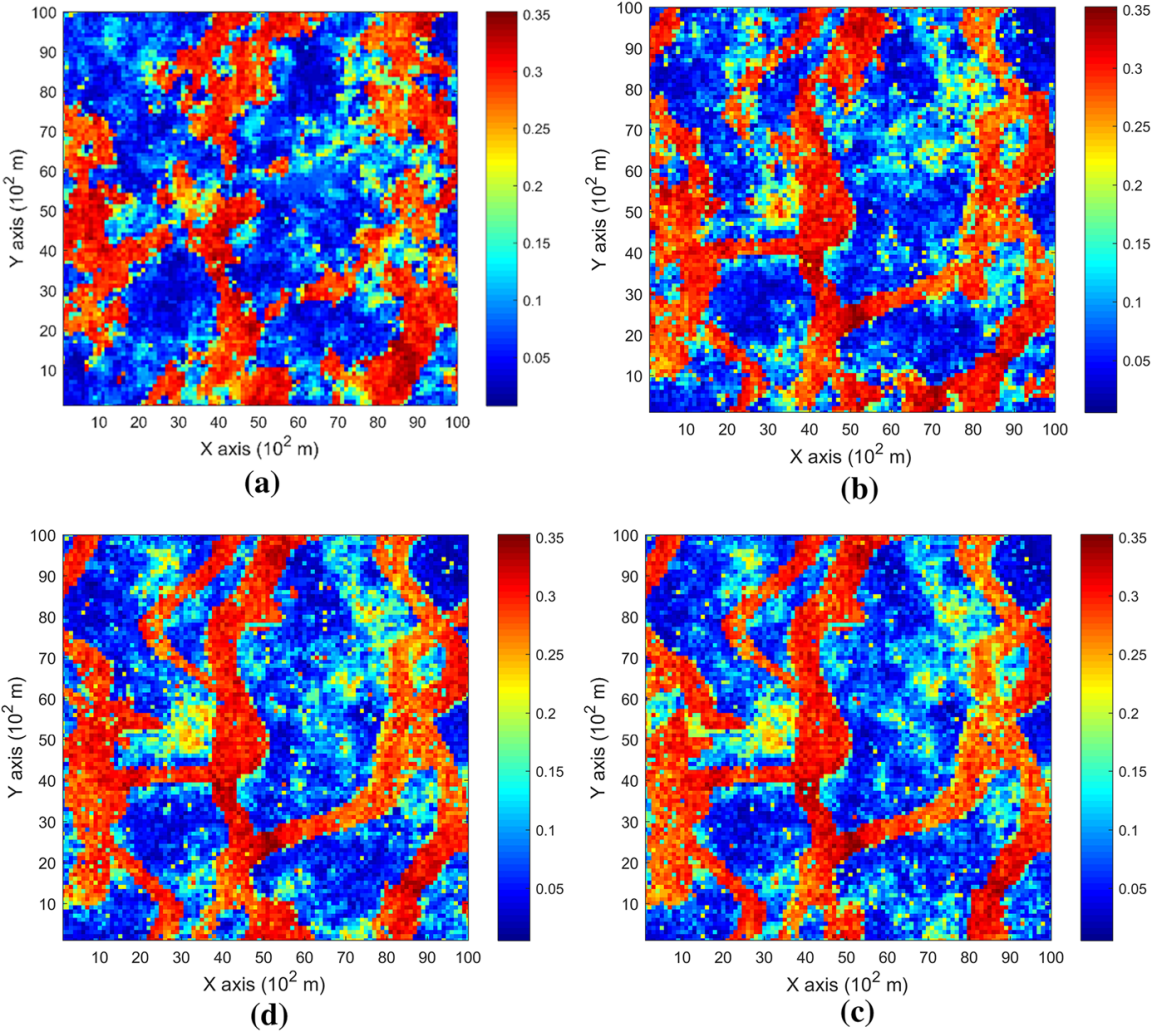
Comparing the realizations of high-order simulation by applying different local neighborhood size, with 200 sample data using the exhaustive data as the TI. The maximum order of Legendre polynomials to approximate the cpdfs is 10 for all the realizations. Realizations with neighborhood of: **a** 6 conditioning data, **b** 12 conditioning data, **c** 20 conditioning data, and **d** 30 conditioning data

**Fig. 15:**
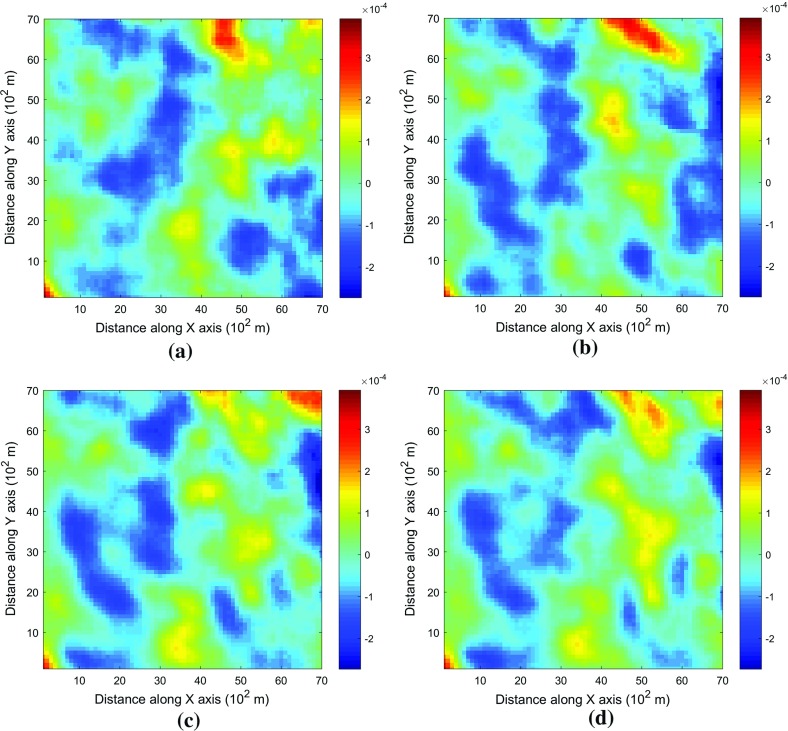
Comparing the third-order cumulant maps of the realizations of the high-order simulation by applying different local neighborhood size, with 200 sample data using the exhaustive data as the TI. The maximum order of Legendre polynomials to approximate the cpdfs is 10 for all the realizations. Third-order cumulant maps of one realization with neighborhood of: **a** 6 conditioning data, **b** 12 conditioning data, **c** 20 conditioning data, and **d** 30 conditioning data

**Fig. 16:**
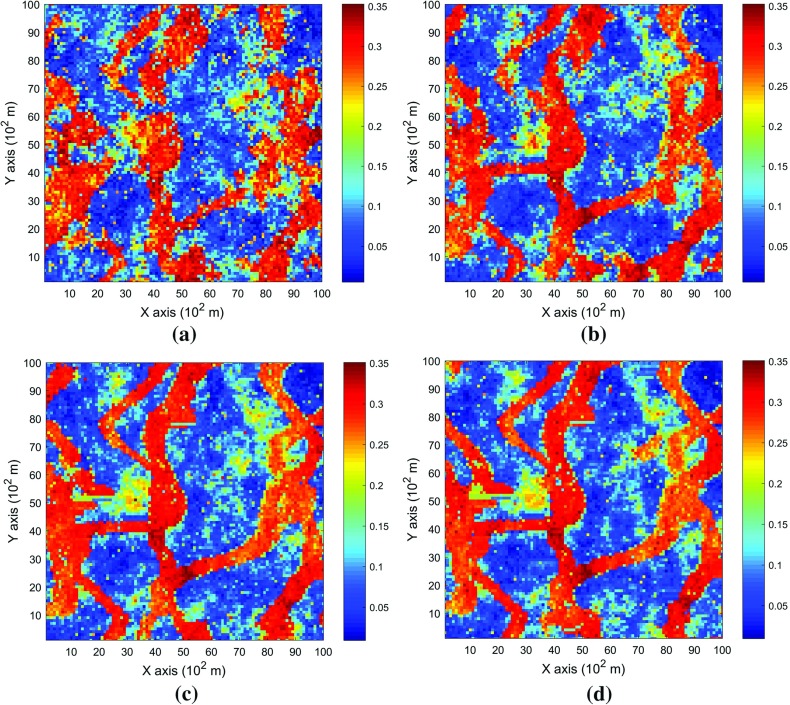
Comparing the realizations of the high-order simulation by applying the different order of truncated Legendre polynomial series, with 200 sample data using the exhaustive data as the TI. The number of conditioning data in the local neighborhood is 12 for all the realizations. Realizations of the high-order simulation by approximating the cpdf with Legendre polynomial series up to: **a** order 6, **b** order 10, **c** order 20, and **d** order 30

**Fig. 17:**
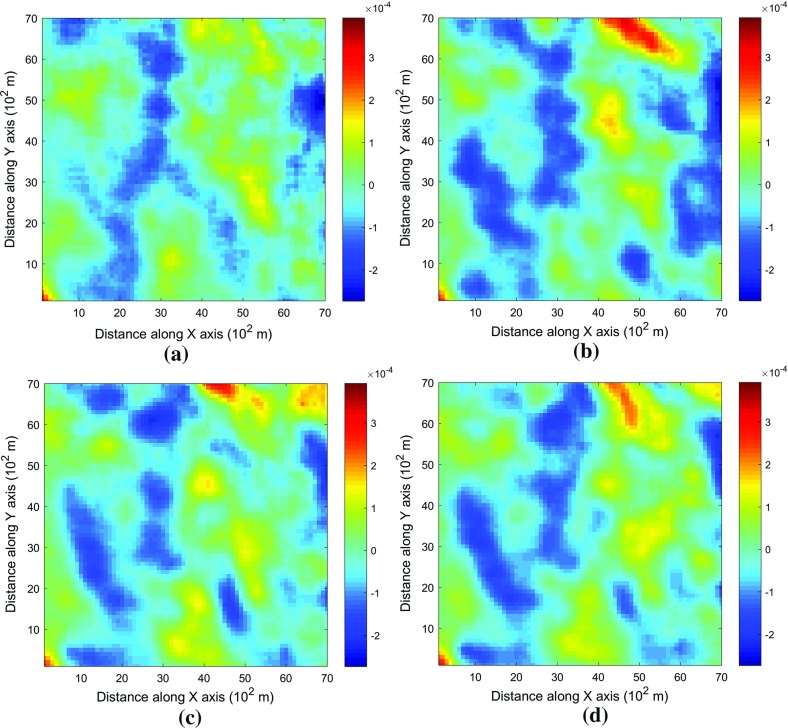
Comparing third-order cumulant maps of the realizations of the high-order simulation by applying the different order of truncated Legendre polynomial series, with 200 sample data using the exhaustive data as the TI. The number of conditioning data in the local neighborhood is 12 for all realizations. Third-order cumulant map of one realization of the high-order simulation by approximating the cpdf with Legendre polynomial series up to: **a** order 6, **b** order 10, **c** order 20, and **d** order 30

For validation and sensitivity analysis, further tests are conducted specifically to demonstrate the impacts of the number of conditioning data and the maximum order of Legendre polynomial series. In order to restrict the effects of the conflicting statistics between the TI and the sample data, the same data set from Example 1 is used to evaluate the sensitivity of the related parameters. The experiments are taken for each individual parameter without considering the possible dependencies between them. In all the experiments, the parameters not being tested remain the same as in Example 1. Furthermore, the random seed used to generate the visiting path is also fixed for all the simulations in the experiments, so that the impact of the different visiting paths is excluded. Figures 14 and 15 depict the realizations of the high-order simulation with different neighborhood sizes and their corresponding third-order cumulant maps. In addition, Figs. 16 and 17 show the realizations of high-order simulation with respect to the order of the Legendre polynomial series in order to approximate the cpdfs (as well as their corresponding third-order cumulant maps). From the results, it can be seen that both the size of the neighborhood and the maximum order of the polynomials have considerable impacts on the high-order simulation results. In particular, using a small size of the neighborhood of 6 grid cells or a Legendre polynomial order less than 6 results in a poor reproduction of the spatial patterns, as well as the cumulant maps. However, when the size of the neighborhood increases to more than 12 or the order of polynomials is greater than 10, the differences become trivial. Although the testing is for a specific data set, and the size neighborhood should be larger in 3D space than in 2D space, it can be expected that a similar sensitivity analysis can be applied to choose the appropriate parameters on a case-by-case basis.

## Conclusions

The main contributions of this paper are as follows. Firstly, starting from the high-order simulation method based on Legendre polynomial series, a new computational model in the form of a unified empirical function is developed to approximate the conditional probability density function (cpdf). The computational model leads to an estimation of the cpdf without calculating the high-order spatial cumulants or moments term by term. As a consequence, it not only greatly reduces the computational requirements, but it also provides a more accurate approximation of the cpdf through Legendre polynomial series in comparison to the previous high-order simulation algorithm based on Legendre cumulants. Secondly, two new algorithms to derive the cpdf and conditional cumulative distribution function (ccdf) based on the above computational model are developed; they both use the properties of Legendre polynomials to simplify the computation and avoid an explicit expansion of a multivariate Legendre series. Lastly, the spatial template used in the current high-order simulation method is dynamically changing with the computation of the probability distribution in real time, without storing data events. In addition, a flexible strategy to search replicates from the training image (TI) is proposed and implemented to deal with the conflicts between the statistics of the sample data and the TI.

Tests show the capacity of the proposed algorithm to reproduce complex geological patterns, and, in addition, that both the overall distribution and the high-order spatial statistics of the data are reproduced by the high-order simulations. Comparing the results of the high-order simulation in different cases with those of filtersim, the high-order simulation outperforms in the reproduction of high-order spatial statistics. This result becomes more notable in cases where there are conflicts in the spatial statistics between the sample data and the TI. This demonstrates that the high-order simulation has a more data-driven nature, whereas the filtersim is more TI-driven. Although the computational cost is significantly reduced (depending on the size of the training image, the number of neighborhoods, and the maximum order of Legendre polynomial series), the simulation is still slower than the filtersim method. However, since the computations of the cpdf are carried out on each replicate with the same type of calculation, the procedure could be parallelized so that the simulation can be further accelerated through parallelization techniques, such as GPU programming. It should also be noted that the approximation of cpdfs by Legendre series or any kind of polynomial series may generate problems of non-positive probability densities; further research is needed to address this issue.

## Appendix: Expansion Series of Probability Density Function Based on the Spatial Legendre Moments

Suppose that the multivariate function $f\left( {z_{0} ,z_{1} , \ldots ,z_{N} } \right)$ is the density function related to the joint distribution of random variables on a spatial template **T**, and that it can be expressed as a Legendre polynomial series. The sequence of Legendre polynomials at different orders forms a set of orthogonal bases of a Hilbert space containing all the continuous functions defined on $D = \left[ { - 1, 1} \right]^{N + 1}$; the inner product is defined as

A.1 $$ \begin{array}{*{20}c} {\langle{g,h}\rangle = \displaystyle\int _{D} ghdz_{0} \ldots dz_{N},} \\ \end{array} $$

where $g,h$ are functions in the Hilbert space.

From the orthogonal property of a Legendre polynomial and the definition of its norm shown in Eq. (7), there is

A.2 $$ \begin{array}{*{20}c} {f\left({z_{0},z_{1}, \ldots,z_{N}} \right) = \mathop \sum \limits_{{w_{0} = 0}}^{\infty} \mathop \sum \limits_{{w_{1} = 0}}^{\infty} \cdots \mathop \sum \limits_{{w_{N} = 0}}^{\infty} \left\langle {f,\bar{P}_{{w_{0}}} \bar{P}_{{w_{1}}} \cdots \bar{P}_{{w_{N}}}} \right\rangle \bar{P}_{{w_{0}}} \bar{P}_{{w_{1}}} \cdots \bar{P}_{{w_{N}}},} \\ \end{array} $$

where the set {$\bar{P}_{{w_{0} }} \bar{P}_{{w_{1} }} \cdots \bar{P}_{{w_{N} }} |w_{i} = 0,1,2, \ldots , 0 \le i \le N\}$ are the orthonormal bases of the Hilbert space and $\bar{P}_{{w_{i}}} \left({z_{i}} \right) = \frac{{P_{{w_{i}}} \left({z_{i}} \right)}}{{||P_{{w_{i}}} ||}}, 0 \le i \le N$ is the normalized Legendre polynomial. Therefore

A.3 $$ \begin{array}{*{20}c} {\bar{P}_{{w_{0}}} \bar{P}_{{w_{1}}} \cdots \bar{P}_{{w_{N}}} = \frac{{\bar{P}_{{w_{0}}} \bar{P}_{{w_{1}}} \cdots \bar{P}_{{w_{N}}}}}{\parallel{P_{{w_{0}}}\parallel \cdots \parallel{P_{{w_{N}}}}}\parallel} = \mathop \prod \limits_{i = 0}^{N} \sqrt {w_{i} + \frac{1}{2}} \cdot P_{{w_{0}}} P_{{w_{1}}} \cdots P_{{w_{N}}}} \\ \end{array} $$

A.4 $$ \begin{array}{*{20}c} \langle{f,\bar{P}_{{w_{0}}} \bar{P}_{{w_{1}}} \cdots \bar{P}_{{w_{N}}}\rangle = \mathop \prod \limits_{i = 0}^{N} \sqrt {w_{i} + \frac{1}{2}} \cdot \langle{f,P_{{w_{0}}} P_{{w_{1}}} \cdots P_{{w_{N}}}}\rangle.} \\ \end{array} $$

Combining Eqs. (A.2)–(A.4), it is seen that

A.5 $$ f\left({z_{0},z_{1}, \ldots,z_{N}} \right)\begin{array}{*{20}l} {= \mathop \sum \limits_{{w_{0} = 0}}^{\infty} \mathop \sum \limits_{{w_{1} = 0}}^{\infty} \cdots \mathop \sum \limits_{{w_{N} = 0}}^{\infty} \mathop \prod \limits_{i = 0}^{N} \left({w_{i} + \frac{1}{2}} \right) \cdot \left\langle {f,P_{{w_{0}}} P_{{w_{1}}} \cdots P_{{w_{N}}}} \right\rangle P_{{w_{0}}} P_{{w_{1}}} \cdots P_{{w_{N}}}.} \\ \end{array} $$

Note that $f\left( {z_{0} ,z_{1} , \ldots ,z_{N} } \right)$ is the pdf; thus

A.6 $$ \begin{aligned}\langle{f,P_{{w_{0}}} P_{{w_{1}}} \cdots P_{{w_{N}}}}\rangle =  \int _{D} P_{{w_{0}}} \left({z_{0}} \right)P_{{w_{1}}} \left({z_{1}} \right) \cdots P_{{w_{N}}} \left({z_{N}} \right)f\left({z_{0},z_{1}, \ldots,z_{N}} \right){\text{d}}z_{0} \cdots {\text{d}}z_{N} \\ = E\left[{h_{1}, \ldots,h_{N};P_{{w_{0}}} \left({z_{0}} \right)P_{{w_{1}}} \left({z_{1}} \right) \cdots P_{{w_{N}}} \left({z_{N}} \right)} \right] . \end{aligned}$$

To use the Legendre polynomials as the bases without normalization and avoid computation of the square roots, the spatial Legendre moments are defined as

A.7 $$ \begin{array}{*{20}c} {L_{{w_{0} w_{1} \cdots w_{N}}}^{\varvec{T}} = \mathop \prod \limits_{i = 0}^{N} \left({w_{i} + \frac{1}{2}} \right) \cdot \langle{f,P_{{w_{0}}} P_{{w_{1}}} \cdots P_{{w_{N}}}}\rangle,} \\ \end{array} $$

which is equivalent to the definition in Eq. (8).

Furthermore, from Eqs. (A.5)–(A.7), one can directly derive the expansion series of the pdf based on the spatial Legendre moments, which appears in Eq. (10). A similar derivation works for the truncated Legendre polynomial series, since the corresponding function space forms a finite-dimensional subspace of the above Hilbert space.
